# Стволовые клетки коры надпочечников: основные сигнальные пути

**DOI:** 10.14341/probl12819

**Published:** 2021-12-30

**Authors:** О. В. Глазова, М. В. Воронцова, Л. В. Шевкова, Н. Сакр, Н. А. Онянов, С. А. Казиахмедова, П. Ю. Волчков

**Affiliations:** Национальный медицинский исследовательский центр эндокринологии; Московский физико-технический институт (национальный исследовательский университет); Национальный медицинский исследовательский центр эндокринологии; Национальный медицинский исследовательский центр эндокринологии; Московский физико-технический институт (национальный исследовательский университет); Московский физико-технический институт (национальный исследовательский университет); Московский физико-технический институт (национальный исследовательский университет); Московский физико-технический институт (национальный исследовательский университет); Национальный медицинский исследовательский центр эндокринологии; Московский физико-технический институт (национальный исследовательский университет)

**Keywords:** надпочечники, стволовые клетки, сигнальные пути, клетки-предшественницы

## Abstract

Стволовые клетки взрослого организма вызывают сегодня большой интерес ввиду активного развития клеточных и геномных технологий. Именно они являются мишенью новых терапевтических подходов, основанных на редактировании мутаций или восполнении органов, поврежденных в результате аутоиммунной реакции, старения и прочих патологических процессов. Также стволовые клетки, в том числе пациент-специфичные (индуцированные плюрипотентные стволовые клетки), и полученные путем дифференцировки из них культуры тканей и органоидов являются наиболее приближенными к in vivo моделями человеческого организма, что позволяет получать более релевантные данные по тестированию различных терапевтических подходов и фармакологических препаратов. В представленном обзоре описаны основные молекулярные пути, ответственные за поддержание гомеостаза коры надпочечников — сложного, структурно и функционально неоднородного органа. Кора надпочечников обновляется в течение онтогенеза организма за счет пула клеток-предшественниц (стволовых клеток и прогениторов), находящихся в тесном контакте с дифференцированными стероидогенными клетками и подвергающихся постоянному контролю эндокринных и паракринных сигналов. Понимание путей сигналинга и взаимодействия разных типов клеток позволит разработать наиболее подходящие протоколы получения клеток коры надпочечников на разных стадиях дифференцировки для использования их в научных и медицинских целях.

## Abstract (en)

Nowadays stem cells of adult type are attractive in case of active development of cell and genome technologies. They are the target of new therapeutic approaches, which are based on correction of mutations or replenishment of organs, that were damaged by autoimmune reactions, aging or other pathological processes. Also stem cells, including patient-specific (induced Pluripotent Stem Cells, iPSCs), and obtained by differentiation from them tissue cultures and organoids are the closest models to in vivo researches on humans, which gives an opportunity to get more relevant data while testing different therapeutic approaches and pharmacological drugs. The main molecular pathways, that are essential for homeostasis of a cortex of a adrenal gland — compound, structurally and functionally heterogeneous organ, is described the presented review. The adrenal cortex is renewing during the organism’s ontogenesis at the expense of the pool of stem and progenitors cells, which are in tight junctions with differentiated steroidogenic cells and which are under constant control of endocrine and paracrine signals. The understanding of signaling pathways and interactions of different cell types will give an opportunity to develop the most suitable protocols for obtaining cells of adrenal gland cortex in a different stages of differentiation to use them in scientific and medical purposes.

## ВВЕДЕНИЕ

Надпочечники — это парные эндокринные органы, расположенные по одному в забрюшинном пространстве, топографо-анатомически над верхними полюсами почек, производящие стероидные гормоны и катехоламины. Железы состоят из двух частей различного эмбрионального происхождения: внешнего слоя — коркового вещества и центральной зоны — мозгового вещества. Кора надпочечников, о которой идет речь в данном обзоре, производит три типа стероидов: минералокортикоиды, глюкокортикоиды и андрогены. Производство гормонов разделено физиологически и регулируется независимыми петлями эндокринной обратной связи: ренин-ангиотензин-альдостероновой системой (РААС) и гипоталамо-гипофизарно-надпочечниковой осью (ГГН), которые контролируют выработку минералокортикоидов и глюкокортикоидов/андрогенов соответственно. Эта функциональная компартментализация отражена гистологически, так как кора разделена на три отдельные гистологические зоны в центростремительном направлении: клубочковая зона (zona glomerulosa, zG), пучковая зона (zona fasciculata, zF) и сетчатая зона (zona reticularis, zR). Кроме того, надпочечник окружен капсулой, содержащей стволовые клетки, под которой в zG располагаются субкапсулярные клетки-прогениторы. Паракринные пути передачи сигналов, поддерживаемые популяциями стволовых клеток/прогениторов, о которых речь пойдет ниже, и включающие гетеротипические клеточные взаимодействия, являются основными детерминантами анатомической и функциональной зональности надпочечников (рис. 1).

**Figure:**
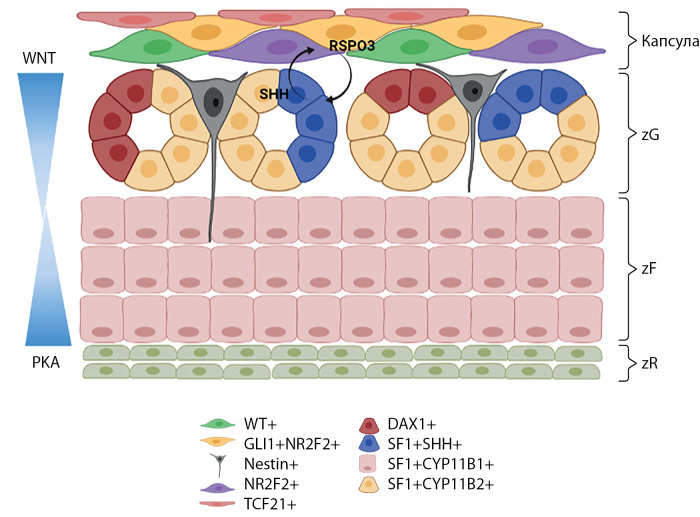
Рисунок 1. Схематичное строение коры надпочечников с указанием основных типов стволовых клеток и прогениторов, локализующихся в капсуле и клубочковой зоне.

На основе изучения стволовых клеток крови (гематопоэтических стволовых клеток, hematopoietic stem cells, HSCs) в научном сообществе сформировался консенсус по использованию терминов, описывающих клеточные состояния разного уровня потентности. Согласно этой терминологии, в данной работе термин «стволовые клетки» будет означать редко делящиеся, длительно сохраняющиеся в состоянии покоя мультипотентные клетки. Также будет использоваться термин «прогениторы», который в более широком смысле описывает уникальные клеточные популяции, способные поддерживать и восполнять в случае резекции необходимое количество специализированных клеток посредством активной пролиферации и дифференцировки [1]. Гомеостатическое поддержание и восстановление коры надпочечников требует тонкой координации между стволовыми клетками и прогениторами. Эти популяции клеток демонстрируют высокий уровень пластичности и по-разному активируются в ответ на различные факторы, включая эмбриональное развитие, физиологические потребности и патологические процессы.

В этом обзоре мы суммируем последние данные о паракринных регуляторных петлях, которые управляют функцией стволовых клеток/прогениторов в коре надпочечников, и их важности для гомеостаза органа на протяжении всей жизни.

## ЭМБРИОНАЛЬНОЕ РАЗВИТИЕ

Для понимания становления и функционирования различных популяций стволовых клеток и прогениторов надпочечников, а также для разработки подходов к получению таких линий in vitro крайне необходимо понимать основные молекулярные процессы, происходящие во время эмбрионального развития органа. Кора надпочечников плода формируется из зачатка надпочечников (adrenogonadal primordium, AGP), который, в свою очередь, происходит из целомического эпителия и лежащей в его основе промежуточной (мезонефрической) мезодермы в течение первых 4–6 нед гестации у людей и на 9,5-й день эмбрионального развития (E9.5) у мышей. На этом этапе инициируется экспрессия фактора транскрипции стероидогенного фактора 1 (SF1, кодируемого геном NR5A1 у людей или Nr5a1 у мышей), запускающего дифференцировку AGP с последующим формированием гонад и коры надпочечников с уникальными стероидогенными программами обоих органов [2]. Для инициации экспрессии адренокортикального Nr5a1 необходим фетальный адреналоспецифический энхансер (FAdE), активный исключительно во время позднего развития AGP [3]. Экспрессия FAdE индуцируется комплексом белков Hox-Pbx-Prep1 и поддерживается этим комплексом вместе с ауторегуляцией самим Sf1. Интересно, что у мышей, дефицитных по гену Pbx, не развиваются надпочечники, но развиваются гонады, в которых экспрессия Sf1 сохраняется, что позволяет предположить, что существует другой фактор и/или энхансер на промоторе Sf1, контролирующий экспрессию последнего в этих тканях [4]. AGP разделяется на зачатки надпочечников и гонад (8-я неделя беременности (E10.5)), и к 9-й неделе беременности (E12.5) клетки нервного гребня проникают в зачаток надпочечников с образованием центрального мозгового вещества надпочечников.

После инкапсуляции и инфильтрации клетками нервного гребня формируется ниша стволовых клеток/прогениторов дефинитивной надпочечниковой коры (коры взрослого типа). В текущем представлении для реализации этого процесса необходимо 4 события:

1. подавление экспрессии Sf1 в фетальной коре, переход части клеток в стволовое состояние и миграция их в капсулу;

2. формирование капсулы из близлежащих мезенхимальных клеток плода (8–9-я неделя беременности у людей, E11.5–E12.5 у мышей);

3. дифференцировка капсулярных клеток и активация экспрессии Sf1 в дефинитивной коре;

4. регрессия фетальной коры. У людей фетальная кора надпочечников регрессирует и в конечном итоге заменяется окончательной (дефинитивной) корой взрослого типа в течение нескольких недель после рождения [5]. Все вышеперечисленные события происходят в строго определенные временные интервалы, что обеспечивает правильное развитие и нормальное функционирование коры надпочечников [6]. Хотя механизмы, регулирующие переход между фетальной корой плода и дефинитивной корой, все еще плохо изучены, исследования показывают, что транскрипционный фактор DAX1 (кодируется геном Nr0b1) и сам SF1 действуют как корепрессоры [7], подавляющие FAdE во время перехода от фетальной к дефинитивной коре [3].

## СТВОЛОВЫЕ КЛЕТКИ И ПРОГЕНИТОРЫ КОРЫ НАДПОЧЕЧНИКОВ ВЗРОСЛОГО ТИПА

Описанный процесс формирования коры надпочечников на разных стадиях эмбриогенеза дает представление об источнике стволовых клеток в надпочечниках взрослого типа — клетках фетальной коры и мезенхимальных клетках, окружающих формирующийся надпочечник. Во взрослом органе описаны различные популяции клеток-предшественниц, локализующихся в надпочечниковой капсуле и коре. Эти капсульные и корковые популяции взаимодействуют через реципрокные сигнальные сети с помощью не до конца изученных механизмов, координируя центростремительную пролиферацию и дифференцировку кортикальных клеток в ответ на паракринную и эндокринную сигнализацию, тем самым поддерживая гомеостаз коры надпочечников. В следующих разделах мы кратко суммируем текущие знания о ключевых популяциях клеток-предшественниц и их участии в паракринных сигнальных сетях, важных для адренокортикального гомеостаза.

Капсула

Капсула толщиной в несколько клеток состоит из нескольких длительно сохраняющихся мультипотентных популяций SF1-отрицательных (SF1-) клеток. Эти популяции отличаются экспрессией разных факторов и белков: белок семейства содержащих домен цинковых пальцев 1 типа GLI (GLI family zinc finger 1, GLI1), гомолог 1 опухолевого белка Вильмса (Wilms tumor suppressor gene 1, WT1), фактор транскрипции 21 (TCF21), R-спондин 3 (R-spondin 3, RSPO3), Yes-ассоциированный белок (Yes-associated protein, YAP), транскрипционный коактиватор с PDZ-связывающим мотивом (transcriptional coactivator with the PDZ-binding motif, TAZ) и нестин (Nestin). Все эти популяции клеток обладают различной пролиферативной способностью и свои особенности взаимодействия с субкапсулярными прогениторами, экспрессирующими SF1 (SF1+).

GLI1

GLI1 является транскрипционным эффектором канонического сигнального пути Hedgehog (HH). В надпочечниках Sonic hedgehog (SHH) секретируется прогениторами zG и передает сигналы капсульным клеткам Gli1+, что приводит к Gli1-зависимой транскрипции канонических генов-мишеней SHH [8–10]. Недавние исследования показывают, что Gli1-зависимая транскрипция может быть активирована посредством SHH-независимых механизмов [11]. Во время эмбрионального развития по крайней мере часть клеток, экспрессирующих Gli1, происходит из клеток FAdE+ [12] и составляет самую большую популяцию клеток в капсуле. Во взрослом органе их потомки мигрируют центростремительно в кору, дифференцируясь в клетки SF1+, подмножество которых также экспрессирует SHH [10]. Примечательно, что у самцов мышей во взрослом возрасте вклад Gli1+ клеток в корковый гомеостаз существенно снижается, но может усиливаться во время регенерации zF [10, 13]. Хотя было замечено, что обновление надпочечников у самок мышей происходит быстрее, чем у самцов [14], недавнее исследование А. Grabek и соавт. [15] показало, что половой диморфизм может частично объясняться андрогенами, ограничивающими вклад Gli1+ клеток в процессы регенерации коры надпочечников особей мужского пола.

WT1

WT1 является регулятором транскрипции SF1 в AGP, но после разделения зачатков надпочечников и гонад экспрессия WT1 в надпочечниках репрессируется [16]. Молекулярная биология WT1 сложна, и по крайней мере 36 различных изоформ белка могут быть получены с помощью комбинации альтернативных сайтов начала транскрипции, альтернативного сплайсинга и редактирования РНК. Альтернативный сплайсинг на стыке экзонов 9 и 10 генерирует изоформы, содержащие (+KTS) или не содержащие (-KTS) три аминокислоты KTS, что приводит к образованию белков с разными биохимическими и биологическими свойствами. Эктопическая экспрессия изоформы Wt1 -KTS достаточна для предотвращения дифференцировки клеток AGP в стероидогенные клетки путем прямой регуляции экспрессии генов Gli1 и Tcf21, что указывает на значимость этой изоформы в эмбриогенезе органа [17]. Капсульные Wt1+ клетки взрослого надпочечника в норме могут давать некоторое количество Sf1+ адренокортикальных клеток, осуществляя таким образом небольшой вклад в корковый гомеостаз [16–18]. Недавно было продемонстрировано, что специфичная для Sf1+ клеток делеция гена Ezh2, роль которого в поддержании коркового гомеостаза будет рассматриваться ниже, приводит к аплазии zF, совпадающей с накоплением Wt1+ клеток. Это явление дает возможность предположить, что в контексте повышенного запроса клетки Wt1 могут рекрутироваться в качестве супрафизиологических предшественников [19].

TCF21

Во время развития надпочечников мыши Tcf21 экспрессируется начиная с E9.5, и на стадии E12.5 Tcf21+ мезенхимальные зародышевые клетки совместно с таковыми WT1+ обволакивают формирующийся надпочечник. Клетки Tcf21+ дают начало как Sf1- капсулярной, так и Sf1+ кортикальной популяциям до формирования функциональной капсулы. После образования капсулы клетки Tcf21+ вносят вклад в развитие надпочечников, давая Sf1- стромальные клетки. Начиная с E14.5 стадии развития экспрессия Tcf21 ограничивается только капсулярными клетками. Во взрослой коре надпочечников эта популяция генерирует только стромальные клетки, включая десмин-положительные гладкомышечные клетки (SMC) и PDGFRA-положительные фибробластные клетки [12] .

RSPO3

RSPO3 — паракринный фактор, высвобождаемый из капсулы надпочечников, действующий как усилитель передачи сигналов канонического пути WNT [20]. Rspo3 экспрессируется в капсуле надпочечников мыши, начиная с E12.5, совместно с клетками Gli1+ и Nr2f2+ (маркер мезенхимальных стволовых клеток). Потеря RSPO3 в зрелом возрасте приводит к истончению коры из-за потери zG, о чем свидетельствует потеря маркеров прогениторов SHH и Wnt4, а также маркеров зональности DAB2 и CYP11B2. Экспрессия капсульного Gli1 у таких животных также нарушается, что согласуется с потерей передачи сигналов SHH [21]. На основе описанных наблюдений была предложена модель двойной паракринной связи между корой и капсулой: капсула поддерживает идентичность и пролиферацию zG за счет секреции RSPO3, тогда как клубочковая зона поддерживает пул капсулярных предшественников за счет секреции SHH.

YAP

Путь передачи сигналов Hippo участвует в контроле размера, обновления и регенерации органов [22]. Два эффектора передачи сигналов Hippo — YAP и TAZ — экспрессируются как в капсуле надпочечников мыши, так и во всей коре надпочечников. Было показано, что SF1-контролируемая делеция одной копии Yap и двух копий Taz приводит к дефектам надпочечников у самцов мышей [23]. Такие мыши демонстрировали незначительное снижение уровня кортикостерона при индукции адренокортикотропным гормоном (АКТГ), увеличение накопления липидов и снижение экспрессии мРНК Shh, Nr0b1 и Gli1 в возрасте 10 нед. Последнее может свидетельствовать о частичном истощении стволовых клеток и прогениторов надпочечников. Примечательно, что избыточная экспрессия YAP1 наблюдалась в опухолях коры надпочечников у детей и ассоциировалась с плохим прогнозом [24]. Любопытно, что гиперактивация YAP и TAZ (которая достигается удалением их негативных регуляторов, киназ LATS1 и LATS2) вызывает надпочечниковую недостаточность при рождении, скорее всего, из-за обширной трансдифференцировки стероидогенных клеток в миофибробласты — хотя этот процесс еще предстоит однозначно доказать [25]. Таким образом, путь Hippo может иметь специфические, но важные функции как во время эмбрионального развития надпочечников, так и в постнатальном поддержании гомеостаза органа.

Нестин (Nestin)

Нестин — белок промежуточных филаментов типа VI, маркирует небольшую обособленную популяцию клеток, происходящих из нервного гребня, разбросанных по капсуле и распространенных в коре надпочечников мышей [26]. Во взрослом надпочечнике потомки Nes+ клеток мигрируют центростремительно к мозговому веществу и демонстрируют способность к дифференцировке в стероидогенные клетки in vivo и in vitro [26]. Интересно, что мыши, подвергшиеся иммобилизационному стрессу, демонстрируют более быстрое истощение этого пула стволовых клеток, чем контрольные мыши, из-за повышенной скорости центростремительной миграции Nes+ клеток к мозговому веществу надпочечника. Поскольку нестин-положительные клетки разрастаются сквозь кору в сторону медуллы, вероятно, они могут выполнять уникальную функцию по организации взаимодействия между этими зонами во время стрессовых реакций [26][27].

Кора

Недавние исследования также выявили субкапсулярный внешний слой клеток коры как место расположения адренокортикальных прогениторов, рекрутируемых в ответ на эндокринные и паракринные факторы. Текущая модель предполагает, что потомки периферических адренокортикальных стволовых клеток дифференцируются и мигрируют центростремительно, подвергаясь апоптозу на кортикомедуллярной границе, тем самым давая начало сперва субкапсулярным прогениторам SHH+, затем терминально или частично дифференцированным CYP11B2+ и CYP11B1+ клеткам zG и zF соответственно. Эти периферические предшественники характеризуются ядерной экспрессией β-catenin, SF1, SHH, DAX1 и отсутствием CYP11B2 [8–10][13][14][28][29]. Далее будет представлен краткий обзор молекулярных процессов, определяющих различные состояния этих корковых популяций.

Сигнальный путь HH

SHH является единственным лигандом сигнального пути HH, экспрессируемым в надпочечниках. SHH секретируется частично дифференцированным подмножеством клеток Sf1+Cy11b2- zG, начиная с E12.5. Эксперименты по отслеживанию клонов клеток на мышах показали, что Shh+ клетки zG дают начало практически всем кортикальным клеткам во время развития и гомеостаза взрослых надпочечников. В настоящее время известно, что клетки SHH+ дифференцируются в клетки CYP11B2+, которые затем мигрируют и дифференцируются в клетки zF CYP11B1+ посредством репрессии сигнального пути WNT и активации сигнального пути ACTH-зависимой протеинкиназы A (PKA) (будет рассмотрено ниже) [9][10][28][30]. У самцов мышей передача сигналов SHH особенно важна во время регенерации zF, во время которой капсулярные Gli1+ и субкапсульные SHH+ клетки являются предшественницами, рекрутируемыми для репопуляции zF и восстановления стероидогенеза. Действительно, фармакологическое ингибирование пути HH ограничивает регенерацию коры надпочечников [13]. Таким образом, во взрослом надпочечнике SHH, секретируемый клетками zG, через рецептор PTCH1 активирует передачу сигналов Hedgehog в капсульных клетках, что приводит к активации транскрипции, опосредованной GLI1.

Сигнальный путь WNT

Путь передачи сигналов WNT участвует в органогенезе, гомеостазе и контроле клеток-предшественниц во многих тканях, включая кору надпочечников. В каноническом сигналинге лиганды WNT, выделяемые одними клетками, связываются с рецепторами на поверхности клеток-мишеней [31], что приводит к транслокации β-катенина из цитоплазмы в ядро, где он соединяется с фактором транскрипции TCF/LEF(T-cell factor/lymphoid enhancer factor) для инициации транскрипции целевых генов [32]. В коре надпочечников передача сигналов WNT зонально распределена, с выраженным ядерным окрашиванием β-катенина в zG и градиентом затухания в направлении верхней zF [30][33–35].

У мышей канонический WNT каскад устанавливается после образования AGP (E9.5) и инкапсуляции (E12.5), что совпадает с формированием дефинитивной коры надпочечников. Генетически опосредованное нарушение концентрации β-катенина в клетках Sf1+ во время органогенеза (после E12.5) приводит к снижению пролиферации кортикальных клеток и полной регрессии надпочечников на E18.5 [35]. Потеря передачи сигналов β-катенина 50% кортикальных Sf1+ клеток приводит к надпочечниковой недостаточности и выраженному истончению коры у мышей в возрасте 15 нед. К 45-недельному возрасту такие мыши демонстрируют полную гистологическую дезорганизацию и истончение коры надпочечников. Описанные наблюдения подтверждают, что каноническая передача сигналов WNT важна как для активности кортикальных предшественников в эмбриональном развитии, так и поддержания гомеостаза взрослого органа [35]. Большинство Shh+ клеток активно секретируют WNT [36] и истощаются в модели капсульного дефицита RSPO3 [21]. Эффективность регенерации zF также зависит от канонического WNT сигналинга [13].

В недавних исследованиях роли лигандов WNT в поддержании канонической передачи сигналов в коре надпочечников особый акцент был сделан на Wnt4. У людей наследственные инактивирующие мутации WNT4 вызывают SERKAL-синдром — состояние, характеризующееся реверсией пола с женского на мужской и надпочечниковой недостаточностью [37]. У мышей в эмбриональном периоде Wnt4 экспрессируется во всех зонах развивающегося надпочечника с E11.5 [34], с E14.5 экспрессия гена ограничивается внешними отделами коры, а у взрослых мышей WNT4 продуцируется клетками zG [33][34]. Дефицит WNT4 ведет к снижению экспрессии Cyp11b2 в надпочечниках и продукции альдостерона. Значимость этого лиганда также подтверждается мышиной моделью, в которой делеция Wnt4 под контролем SF1 приводит к снижению экспрессии канонических генов-мишеней пути WNT [30].

DAX1

Белок DAX1 (кодируемый геном NR0B1) является эффектором сигнального пути WNT и корепрессором SF1-опосредованной транскрипции [1]. Мутации в DAX1 вызывают врожденную Х-сцепленную гипоплазию надпочечников у людей, которая часто проявляется надпочечниковой недостаточностью [38–40]. Интересно, что DAX1-дефицитные стареющие мыши демонстрируют фенотип, частично напоминающий потерю экспрессии β-катенина: гипофункциональные диспластические надпочечники с потерей пролиферации, что указывает на важность DAX1 в поддержании пула адренокортикальных предшественников [41].

Сигнальный путь PKA (Protein kinase A)

Система обратной связи ГГН регулирует выработку глюкокортикоидов и андрогенов корой надпочечников. Помимо стимуляции выработки стероидов, активация ГГН-оси оказывает митогенное действие на кору надпочечников. Так, АКТГ стимулирует высвобождение кортизола (или кортикостерона у мышей) из zF надпочечников посредством связывания рецептора меланокортина-2 и вспомогательного белка меланокортина (MRAP, melanocortin 2 receptor accessory protein) и активации сигнального пути PKA [42]. У мышей дефицит MRAP приводит к неонатальной летальности, которая устраняется введением экзогенных глюкокортикоидов. У выживших без MRAP наблюдаются постнатальное нарушение стероидогенеза, утолщенная и гиперпластическая капсула, повышенная экспрессия Shh и накопление клеток-предшественниц с активацией WNT сигналинга. Эти данные указывают на критическую роль АКТГ в стимулировании адренокортикальных стволовых клеток/прогениторов к дифференцировке в стероидогенные клетки zF. Кроме того, конститутивная активация PKA-сигналинга в коре надпочечников ингибирует канонический путь WNT, способствуя дифференцировке в zF [30][43].

М. Mathieu и соавт. разработали мышиную модель с SF1-контролируемой делецией гена белка Ezh2, ответственного за метилирование H3K27me3 [19]. Экспрессия Ezh2 в коре надпочечников взрослых мышей в значительной степени ограничивается пролиферирующими клетками на границе zG/zF. В описываемом исследовании у мутантных мышей наблюдалось развитие первичной глюкокортикоидной недостаточности с серьезными дефектами зонирования, в частности аплазией zF и дезорганизацией zG. Интересно, что WNT-сигналинг не изменялся, что позволяет предположить, что действие белка EZH2 в первую очередь относится к клеткам zF. Действительно, этой же группой исследователей было показано, что адренокортикальный EZH2 метилирует промоторы генов, кодирующих негативные регуляторы передачи сигналов PKA. Полученные данные указывают на то, что EZH2 является критическим для направления дифференцировки в клетки zF в ответ на индукцию АКТГ и согласуются с недавними наблюдениями, что экспрессия Ezh2 резко возрастает во время пролиферативного всплеска, сопровождающего регенерацию zF [13]. В совокупности вышеизложенные данные подтверждают важную роль EZH2 в обеспечении zF новыми клетками в ответ на АКТГ во время развития и обновления коры надпочечников.

## ЗАКЛЮЧЕНИЕ

В последние годы были достигнуты многочисленные успехи в изучении молекулярных аспектов функционирования надпочечников. Было показано, что поддержание и обновление стероидогенных клеток в коре надпочечников у взрослых организмов регулируются сложными процессами, в которых задействованы как паракринные, так и эндокринные механизмы. В этом обзоре мы сосредоточились в основном на паракринной коммуникации между капсулой и субкапсулярной корой. Однако в будущем научному сообществу предстоит большая работа по более тонкому описанию известных клеточных популяций, а также поиску новых типов предшественников и альтернативных путей обновления zF. Также внимание ученых все больше сосредотачивается на роли нестероидогенных клеток коры надпочечников, включая компартменты стромальных, иммунных и эндотелиальных клеток, в регуляции рекрутирования предшественников и поддержания клеточного гомеостаза надпочечников. Во многом эти задачи будут решаться с использованием новых методов, в частности секвенирования транскриптомов единичных клеток. Этот подход позволит лучше понять устройство надпочечников людей, так как позволяет определять клеточные популяции в образцах, полученных после хирургических вмешательств. Более того, хотя половой диморфизм в коре надпочечников известен давно, только в последние несколько лет становятся понятными механизмы влияния андрогенов на обновление тканей надпочечников. Понимание этого влияния необходимо для объяснения связанной с половой принадлежностью специфики патогенеза, которая характерна для многих заболеваний надпочечников.

Новые знания о тонкой молекулярной структуре типов клеток надпочечников могут быть использованы в будущем для разработки протоколов получения стероидогенных клеток и клеток-предшественниц для клеточной и генной терапии. На сегодняшний день разрабатываются in vitro подходы по получению пациент-специфичных стероидогенных клеток, способных компенсировать недостаток стероидогенеза при врожденной дисфункции коры надпочечников путем экзогенной экспрессии SF1 [44]. Однако такие клетки, во-первых, могут иметь смешанный гонадо-адренальный фенотип из-за активации всех или почти всех генов-мишеней этого транскрипционного фактора, и в таком случае клеточная терапия может приводить к нежелательным побочным эффектам. Во-вторых, продолжительность жизни таких клеток, как и почти любых терминально дифференцированных клеток, ограничена. Следовательно, терапевтический эффект будет иметь временный характер. Решением этих проблем может быть получение частично коммитированных клеток-предшественниц, которые будут способны интегрироваться в структуру взрослого органа и дифференцироваться in vivo. Подобные подходы уже применяются в частности для получения прогениторов дофаминергических нейронов [45], что дает основания рассчитывать на успех стратегии и для клеточных линий надпочечников.

## ДОПОЛНИТЕЛЬНАЯ ИНФОРМАЦИЯ

Источники финансирования. Исследование выполнено с использованием средств государственного бюджета по госзаданию № 121030100031-0 от 02.03.2021.

Конфликт интересов. Авторы декларируют отсутствие явных и потенциальных конфликтов интересов, связанных с содержанием настоящей статьи.

Участие авторов. Глазова О.В. — по критерию 1 — основной исполнитель, создание концепции, подбор литературы; по  критерию  2 — подготовка основного текста; Воронцова М.В. — по критерию 1 — научное руководство, по критерию 2 — внесение в рукопись важных правок; Шевкова Л.В. — по критерию 1 — вклад в дизайн текста, по критерию 2 — внесение в рукопись важных правок, оформление; Сакр Н. — по критерию 1 — вклад в дизайн текста, по критерию 2 — внесение в рукопись важных правок; Онянов Н.А.  — по критерию 1 — вклад в концепцию текста, по критерию 2 — внесение в рукопись важных правок; Казиахмедова С.А. — по критерию 1 — вклад в концепцию текста, по критерию 2 — внесение в рукопись важных правок; Волчков П.Ю. — по критерию 1 — научное руководство, по критерию 2 — внесение в рукопись важных правок. Все авторы одобрили финальную версию статьи перед публикацией, выразили согласие нести ответственность за все аспекты работы, подразумевающую надлежащее изучение и решение вопросов, связанных с точностью или добросовестностью любой части работы.
